# *Mycobacterium tuberculosis* Infection of Dendritic Cells Leads to Partially Caspase-1/11-Independent IL-1β and IL-18 Secretion but Not to Pyroptosis

**DOI:** 10.1371/journal.pone.0040722

**Published:** 2012-07-24

**Authors:** Hana Abdalla, Lalitha Srinivasan, Swati Shah, Katrin D. Mayer-Barber, Alan Sher, Fayyaz S. Sutterwala, Volker Briken

**Affiliations:** 1 Department of Cell Biology and Molecular Genetics, University of Maryland, College Park, Maryland, United States of America; 2 Maryland Pathogen Research Institute, University of Maryland, College Park, Maryland, United States of America; 3 Laboratory of Parasitic Diseases, National Institute of Allergy and Infectious Diseases, National Institutes of Health, Bethesda, Maryland, United States of America; 4 Inflammation Program, Department of Internal Medicine, University of Iowa, Iowa City, Iowa, United States of America; 5 Veterans Affairs Medical Center, Iowa City, Iowa, United States of America; University of Louisville, United States of America

## Abstract

**Background:**

Interleukin-1β (IL-1β) is important for host resistance against *Mycobacterium tuberculosis* (Mtb) infections. The response of the dendritic cell inflammasome during Mtb infections has not been investigated in detail.

**Methodology/Principal Findings:**

Here we show that Mtb infection of bone marrow-derived dendritic cells (BMDCs) induces IL-1β secretion and that this induction is dependent upon the presence of functional ASC and NLRP3 but not NLRC4 or NOD2. The analysis of cell death induction in BMDCs derived from these knock-out mice revealed the important induction of host cell apoptosis but not necrosis, pyroptosis or pyronecrosis. Furthermore, NLRP3 inflammasome activation and apoptosis induction were both reduced in BMDCs infected with the *esxA* deletion mutant of Mtb demonstrating the importance of a functional ESX-1 secretion system. Surprisingly, caspase-1/11-deficient BMDCs still secreted residual levels of IL-1βand IL-18 upon Mtb infection which was abolished in cells infected with the *esxA* Mtb mutant.

**Conclusion:**

Altogether we demonstrate the partially caspase-1/11-independent, but NLRP3- and ASC- dependent IL-1β secretion in Mtb-infected BMDCs. These findings point towards a potential role of DCs in the host innate immune response to mycobacterial infections via their capacity to induce IL-1β and IL-18 secretion.

## Introduction

The inflammasome is a multiprotein complex that initiates the maturation of pro-IL-1β and pro-IL-18 to their secreted products via the activation of caspase-1. The inflammasome consists frequently of three principle components: a NOD-like Receptor (NLR) that is a pattern recognition receptor (PRR), the adaptor protein ASC (apoptotic speck-containing protein with a CARD) and the inactive pro-caspase-1 protein [1], [2]. NLRs that are associated with inflammasome signaling include NRP1 (Nalp1/DEFCAP/NAC/CARD1/CLR17.1), NLRP3 (Nalp3/cryopyrin/ CIAS1/PYPAF1/CLR1.1), NLRC4 (IPAF/CARD12/CLR2.1/CLAN) and NOD2 [2], [3]. The sensing of either pathogen associated molecular patterns (PAMP) or danger associated molecular patterns (DAMP) by these cytosolic PRR leads to assembly of active inflammasome and the generation of activated caspase-1 [1], [2].

Inflammasome activation not only leads to cytokine secretion but may also cause pyroptosis, a specific form of cell death, that combines characteristics of necrotic and apoptotic death pathways [4], [5]. Apoptotic caspases (e.g. caspase-3, -8) are not involved in pyroptosis but instead activation of the inflammatory caspase-1 is a defining feature of this death pathway [5], [6]. Furthermore, pyroptosis results in cell lysis via the caspase-1-dependent formation of plasmamembrane pores leading to leaking of cytosolic cellular components [6], [7], [8], [9]. Finally, the cleavage of chromosomal DNA is associated with pyroptosis but is not mediated via caspase activated DNase activation and thus does not produce the characteristic DNA fragmentation pattern associated with apoptotic cell death [6], [10].

*Mycobacterium tuberculosis* (Mtb) is a human pathogen that causes about 10 million cases of tuberculosis resulting in 1–2 million deaths annually [11]. Mtb is a facultative intracellular pathogen which has evolved to manipulate the infected host cell in multiple ways [12], [13], [14], [15], [16]. The inflammasome was proposed to play an important role in host defense against Mtb since mice deficient in IL-1receptor (IL-1RI), IL-1β or IL-18 are more susceptible to infection with Mtb [17], [18], [19], [20], [21]. In bone-marrow derived macrophages (BMDM) and the human macrophage-like cell line, THP-1, the Mtb-mediated induction of IL-1β secretion is dependent upon host cell NLRP3, ASC and Caspase-1 but independent of NLRC4 [20], [22], [23], [24], [25]. Interestingly, these *in vitro* observations are not recapitulated *in vivo*, since total IL-1β levels in the lungs of Mtb-infected *Asc* and *caspase-1/11* knock-out mice were not significantly different from wild-type mice and consistent with this result these mouse strains were less susceptible to Mtb-infection when compared to IL-1β- deficient mice [20], [23]. Thus *in vivo* there are other methods of processing and secreting IL-1β that do not depend upon inflammasome activation. One potential mechanism for the generation of mature IL-1β *in vivo* could thus involve other cell types besides macrophages. Indeed, to date, only the interaction of macrophages with mycobacteria has been analyzed in detail with regard to inflammasome activation.

It is well established that IL1-β is of great importance for host defense against Mtb infections and thus it is important to understand how production of this cytokine is regulated in response to mycobacterial infections. Different roles for inflammasome activation in monocyte and macrophage mediated IL-1β processing and secretion have been described [26], [27]. Alveolar dendritic cells are host cells for Mtb *in vivo* underscoring their potential importance for host defense [28], [29], [30], [31].To date no detailed analysis on the interaction of Mtb with host cell inflammasome and its implication for host cell death has been performed for dendritic cells. An important recent report by the Ehlers group focused on the *in vivo* importance of NLRP3 for host resistance to Mtb infections than a detailed analysis of Mtb-DC interaction [32]. Nevertheless, *in vitro*, they demonstrated that NLRP3 is important for Mtb-induced IL-1β production [32].

Thus we performed a systematic analysis the role of inflammasome components in DC-mediated IL1-β secretion and host cells death induction after Mtb infection. We demonstrate that BMDCs depend on ASC and NLRP3 but not NLRC4 or NOD2 for IL-1β secretion, which is similar to the inflammasome requirements in macrophages. Interestingly, the commonly used caspase-1 knock-out mice are actually also deficient in caspase-11 expression, hence these mice will be referred to as *Casp1/11^−/−^* [33]. The caspase-1/11-deficient BMDCs had only a partial defect in IL-1β and IL-18 secretion. As shown in BMDMs, the BMDCs mediated IL-1βsecretion was dependent on the presence on the mycobacterial ESX-1 secretion system. Finally, BMDCs responded with strong apoptotic but not pyroptotic or pyronecrotic cell death in response to mycobacterial infections that was also dependent upon ESX-1 protein secretion.

## Results

### Inflammasome activation in DCs is partially dependent upon mycobacterial ESX-1 secretion system

Alveolar macrophages are host cells for Mtb in the lungs of infected animals and humans. Inflammasome activation by Mtb in macrophages has been extensively investigated [20], [22], [23], [24], [34], [35], [36] and a role of the ESX-1 secretion in cytokine response has been demonstrate [35], [37]. An important percentage of infected cells in the lungs of mice are dendritic cells and their inflammasome response to Mtb infection has not been investigated in detail [28], [29].

**Figure 1:**
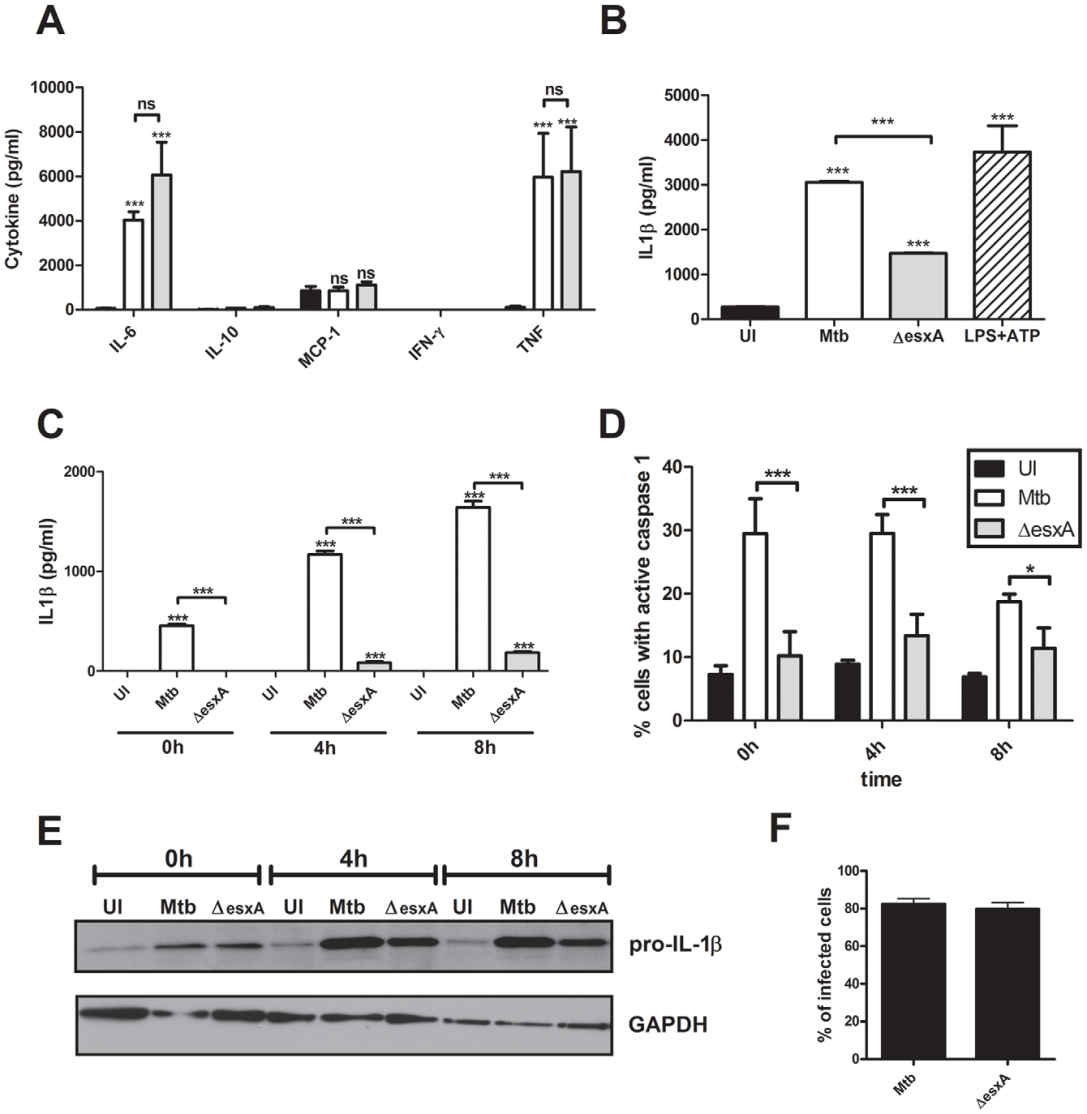
The ESX-1 secretion system of Mtb does not affect secretion of proinflammatory cytokines in dendritic cells but is important for complete inflammasome activation. BMDCs were left uninfected (UI), infected with wild-type Mtb (Mtb) or the esxA deletion mutant (ΔesxA) for 4 h at MOI of 10, washed and incubated for an additional 24 h (A+B) or the indicated timepoints (C+D). (A) The cytokine profile in the supernatants was analyzed using a bead-based immunoassay (black =  uninfected, white =  Mtb, gray =  ΔesxA). (B) and (C) IL-1β secretion was analyzed by ELISA. (D) The percent of cells with activated caspase-1 was determined via flow cytometry using fluorescent caspase-1 substrates (FLICA). (E) Pro-IL-1β protein levels in BMDCs. (F) GFP-labeled bacteria were used to infect BMDCs and rate of infection was determined via flow cytometry. Shown are means and standard deviation of triplicate measurements of one representative experiment out of three. In all figures, the asterisks denote range of p values (* = p<0.05, ** = 0.01>p>0.001,***p<0.001, ns =  not significant ) as determined by one way ANOVA with Tukey's post test.

First, the inflammatory cytokine profile of dendritic cells upon infection with Mtb and the *esxA* deletion mutant of Mtb (MtbΔesxA ) were investigated using a bead-based immunoassay. Both strains induced a significant secretion of the pro-inflammatory cytokines IL-6 and TNF from negligible amounts (<0.1ng/ml) in the supernatants of uninfected cells to 4-6ng/ml in infected cells. There was no induction of IL-10, MCP-1 or IFN-γ secretion by DCs after infection by either strain (Fig. 1A). Next the activation of the inflammasome was investigated via the detection of secreted IL-1β. DCs infected with wild-type Mtb secreted about 3ng/ml of IL-1β which was very similar to the positive control (LPS plus ATP treatment). Interestingly, Mtb deficient in functional ESX-1 secretion system induces less IL-1β secretion of approximately 1.5ng/ml in wild-type BMDCs (Fig. 1B). The secreted IL-1β was the mature form because these supernatants were devoid of pro-IL-1β as tested by a pro-IL-1βspecific ELISA (data not shown). Next we demonstrated if these differences in IL-1β secretion can be detected already right after the 4h infection period and continued to persist during the 4 h and 8 h post infection timepoints(Fig. 1C). To test more directly if the ESX-1 complex was involved in inflammasome activation, we analyzed the activation of caspase-1 via fluorescent substrates and flow cytometry. We could show that only Mtb induced significantly more caspase-1 activation (∼30% positive cells) when compared to uninfected and Mtb *esxA* mutant infected cells (∼8–12%) at 0h and 4h post infection (Fig. 1D). The induction of pro-IL-1β production is very similar right after the infection period as determined by western-blotting (Fig. 1E). At 4 hpi and 8 hpi the pro-IL-1β levels were consistently lower in *esxA* mutant infected cells (Fig. 1E). The rate of infection of BMDCs after 4h is around 80% for both wild-type and mutant Mtb as measured by flow cytometry after infection with GFP-expressing bacteria (Fig. 1F). These results suggest that the inflammasome of DCs is activated by Mtb infection in a manner that is partially dependent upon ESX-1 secreted proteins.

### Apoptosis induction in DCs is partially dependent upon ESX-1

**Figure 2:**
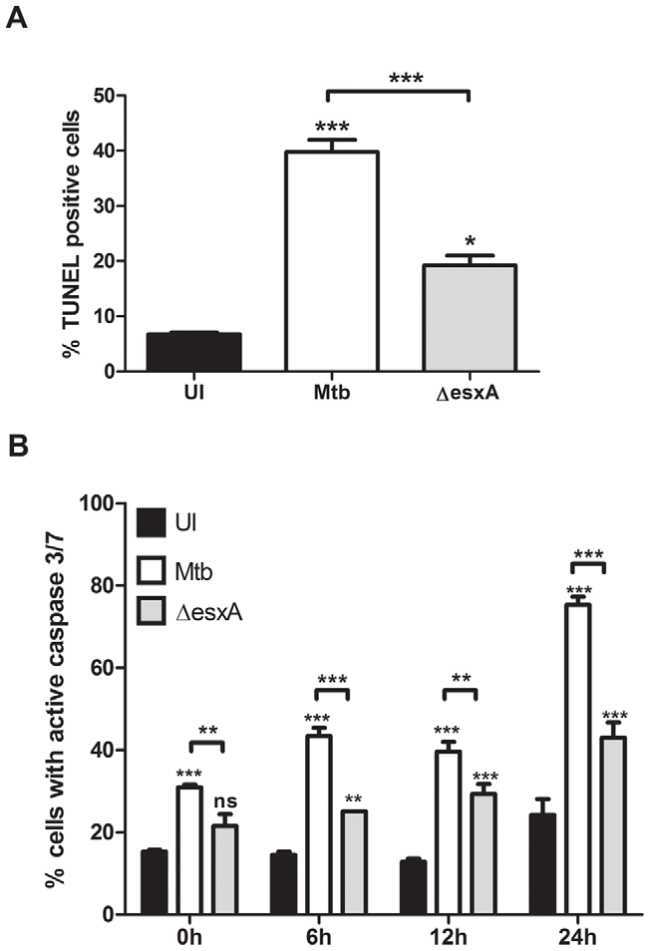
The ESX-1 secretion system of Mtb mediates induction of apoptotic host cell death. (A) BMDCs were left uninfected (UI), infected with wild-type Mtb (Mtb) or the *esxA* deletion mutant (ΔesxA) for 4h at MOI of 10, washed and incubated for an additional 24 h. Cells were harvested and stained using the TUNEL assay. The percent of TUNEL positive cells was determined by flow cytometry. (B) BMDCs were infected as described for (A) and then chased for the indicated time periods. The percentage of cells with active caspase-3/7 was detected as described in material and methods. Shown are means and standard deviation of triplicate measurements of one representative experiment out of three.

The ESX-1 secretion system causes the induction of host cell death upon infection in macrophages [38]. In order to address the role of this secretion system in infected dendritic cells, the cell death induction was first measured via an increase in genomic DNA fragmentation by using the TUNEL assay. The percentage of TUNEL positive cells was determined via flow cytometry after 24 h of infection. The background level of cell death in uninfected cells was about 7% which increased to about 40% in Mtb-infected cells (Fig. 2A). This cell death induction was significantly decreased in the *esxA* mutant to about 20%. The activation of the executioner caspase-3/7 is a key step in apoptotic cell death signaling and thus we examined the activation of these caspases in BMDCs. The percentage of cells with activated caspase-3/7 was quantified via flow cytometry measurement of the caspase-3/7-activated fluorescent substrate (Fig. 2B). The BMDCs infected with Mtb demonstrated a gradual increase from about 30% of caspase-3/7 activated cells right after the 4 h infections to almost 80% of cells 24 h later (Fig. 2B). The background caspase-3/7 activation observed in uninfected cells during the first 12 h remained at 15% and only at 24h increased to about 25%. BMDCs infected with the MtbΔesxA demonstrated significantly less caspase-3 activation when compared to Mtb-infected cells for all the timepoints analyzed (0, 6, 12 and 24 h post-infection)(Fig. 2B). The results demonstrated that the ESX-1 system is involved in host cell death induction in DCs. The differences in BMDC inflammasome activation and cell death induction between Mtb and the *esxA* mutant are not due to difference in uptake as demonstrated in figure 1F.

### ASC and NLRP-3 dependent but partially Caspase-1/11-independent IL-1βproduction in DCs

**Figure 3:**
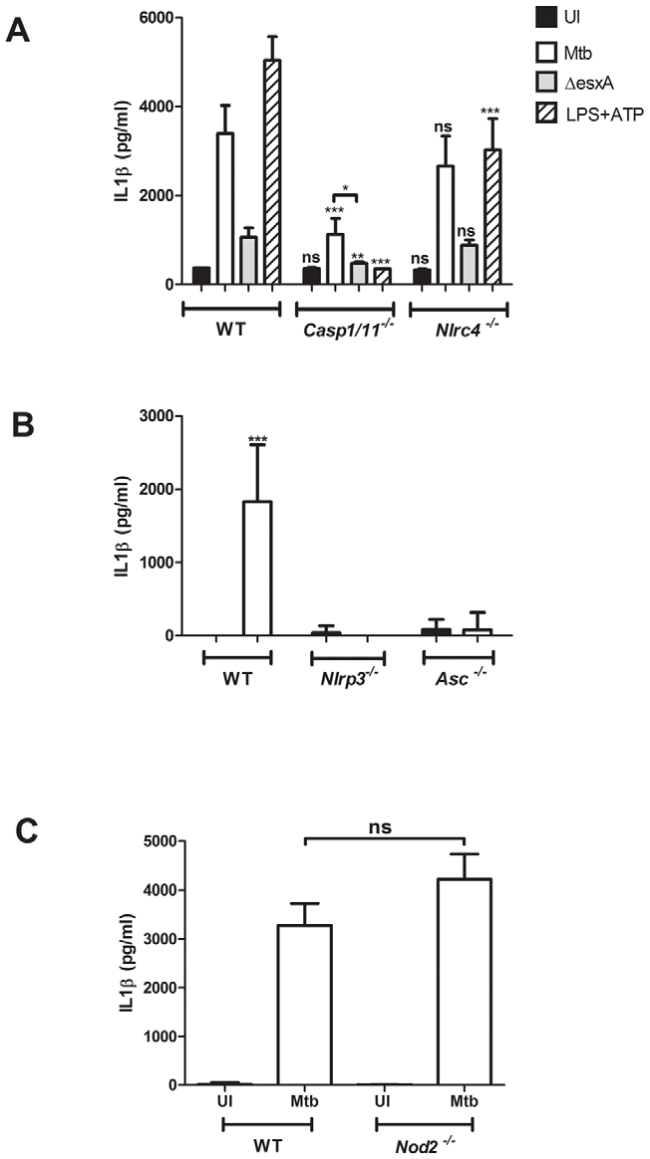
BMDC inflammasome activation upon Mtb infection is dependent upon host cell caspase-1/11, NLRP3 and ASC but not NLRC4 and NOD-2. BMDCs were derived from wild-type (WT), *Casp1/11^−/−^* and *Nlrc4^−/−^* mice (A), WT, *Nlrp3^−/−^* and *Asc^−/−^* mice (B) or WT and NOD-2 mice (C) and infected with Mtb (black bar), ΔesxA (gray bar), left uninfected (UI, black bar) or treated with LPS and ATP (striped bar) as a positive control. The supernatants were harvested after 24 h and the amount of secreted IL-1β was detected by ELISA. Shown are means and standard deviation of triplicate measurements of one representative experiment out of three.

In order to address which inflammasome components are important for Mtb-mediated induction of IL-1β in DCs, cells from various knock-out mouse strains were prepared and infected. The recent publication by the Dixit group demonstrated that the previously generated caspase-1 knock-out mice are actually also deficient in caspase-11 expression, hence the mice will be referred to as *Casp1/11^−/−^* [33]. BMDCs from *Casp1/11^−/−^* mice induced significantly less IL-1β secretion than wild-type mice derived cells (Fig. 3A). Nevertheless, there was still a significant increase in residual IL-1β secretion that was thus independent of caspase-1 and -11 activity in these DCs. Interestingly, this IL-1β secretion was completely lost in *Casp1/11^−/−^* BMDCs infected with the *esxA* deletion mutant. The detected IL-1β was the mature form because pro-IL-1β-specific ELISA failed to detect any significant amount of immature IL-1β (data not shown). The LPS+ATP control treatment also demonstrated that this mechanism of inflammasome activation was completely dependent on caspase-1 and/or -11 activity (Fig. 3A). BMDCs derived from *Nlrc4^−/−^* mice responded with similar IL-1β secretion upon wild-type and ΔesxA Mtb infection when compared to wild-type BMDCs (Fig. 3A). In contrast, BMDCs from ASC- and NLRP3-deficient mice did not activate the inflammasome upon Mtb infection as demonstrated by a lack of increase in IL-1β secretion (Fig. 3B). We furthermore determined that the noninflammatory NLR, NOD2, does not participate in Mtb-mediated inflammasome activation (Fig. 3C) which is in contrast to a NOD2-dependent IL-1β secretion in human monocytes [39].

### Mtb induces apoptosis but not pyroptosis or pyronecrosis in DCs

**Figure 4:**
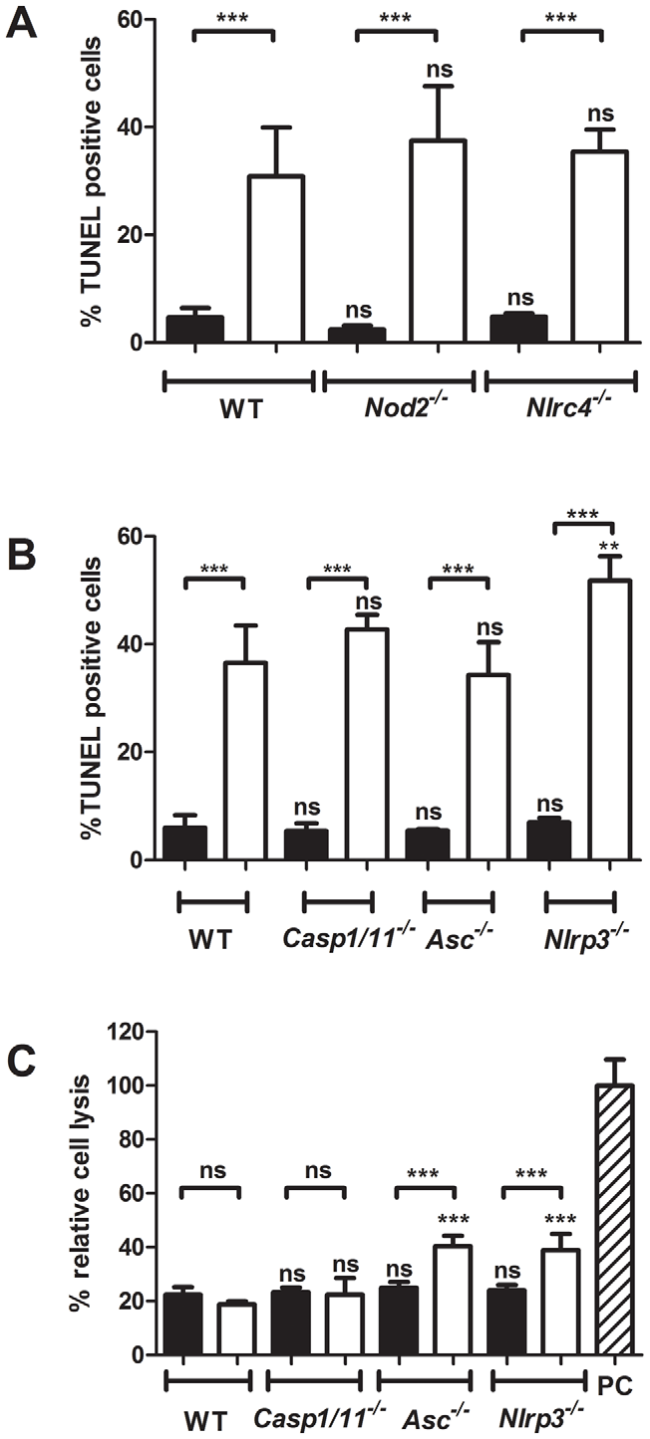
The inflammasome is not involved in BMDC host cell death induction after Mtb infection. BMDCs from indicated wild-type or knockout mouse strains were infected with Mtb (white bars) or left uninfected (black bars). After 24 h the amount of TUNEL positive cells was determined in (A) and in (B). In (C) the amount of necrosis was determined via analysis of the release of adenylate kinase into the supernatant of infected cells relative to detergent lysed cells (PC). Shown are means and standard deviation of triplicate measurements of one representative experiment out of three.

**Figure 5:**
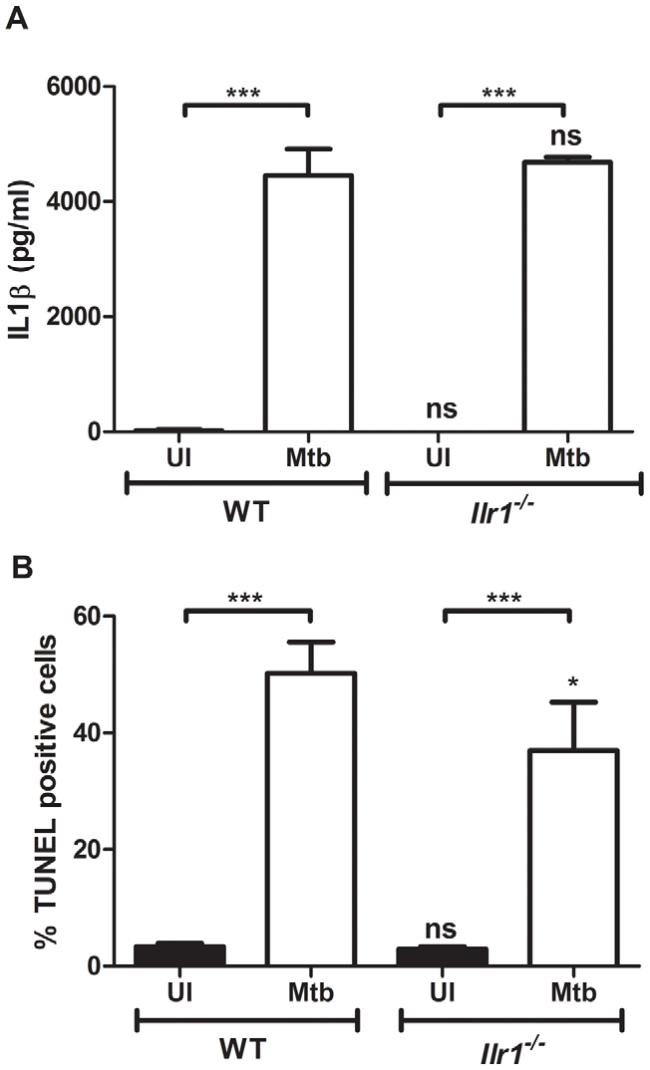
The IL-1 receptor is not necessary for Mtb mediated IL-1β secretion and has a minor role in cell death induction. BMDCs from wild-type (WT) or IL-1RI deficient mice (*Il1r1^−/−^*) were infected with Mtb (white bars) or left uninfected (UI, black bars) and analyzed for IL-1β secretion in (A) and TUNEL staining in (B). Shown are means and standard deviation of triplicate measurements of one representative experiment out of three.

Inflammasome activation is a defining feature of pyroptosis (caspase-1-dependent) and pyronecrosis (ASC-dependent) [4]. We investigated if the Mtb-infected BMDCs undergo one of this forms of cell death. Pyroptosis also leads to disruption of the cell membrane and release of cytosolic proteins into the extracellular environment, whereas apoptotic cell death maintains the membrane integrity [4], [5]. The permeabilization of the cell membrane of infected or uninfected BMDCs was analyzed by the detection of the cytosolic enzyme, adenylate kinase (AK), in the cell supernatant. First, the percentage of TUNEL positive cells in BMDCs derived from *Nlrc4^−/−^* and *Nod2^−/−^* was measured and found to be not significantly different from wild-type BMDCs which was as expected since these cells did not show any difference in inflammasome activation (Fig. 4A.). As shown in Figure 3, the BMDCs of the Caspase-1/11, ASC- and NLRP3- deficient mice all had a clear defect in inflammasome activation after Mtb infection and thus they were now compared for their propensity to undergo cell death by measuring DNA fragmentation (TUNEL staining) and cell membrane disruption (AK release assay) (Fig. 4B and 4C, respectively ). The induction of TUNEL positive cells by Mtb was similar, about 40%, in BMDCs of all the different mouse strains, except for the *Nlrp3^−/−^* cells in which the level of TUNEL positive cells increased to about 50% after 24h of infection (Fig. 4B). These results demonstrate that none of the inflammasome components is important for the cell death induction as measured by DNA degradation. Furthermore, Mtb infection did not lead to an increase in cell membrane permeability after infection of wild-type and *Casp1/11^−/−^* BMDCs. Infected and uninfected BMDCs from these mice had about 20% cell lysis relative to the detergent lysis control, PC, which was set to 100% lysis (Fig. 4C). Surprisingly, we did detect a significant increase of cell lysis in BMDCs of *Asc^−/−^* and *Nlrp3^−/−^* BMDCs which increased in both cases from about 20% in uninfected cells to about 40% in Mtb-infected cells. These results would suggest that these inflammasome components may help to prevent necrosis induction under some circumstances. In conclusion, these results underscored the lack of pyroptosis and pyronecrosis induction in BMDCs after Mtb infection. Furthermore, *Il1r1^−/−^* BMDCs did not demonstrate any defect in IL-1β secretion after 24 h of infection with Mtb, since in wild-type and knock-out BMDCs about 4ng/ml of IL-1β were secreted (Fig. 5A). The IL-1RI-deficient cells showed a statistically significant reduction in TUNEL staining over the same time period with 50.25% in Mtb infected wild-type cells and 37.00% in Mtb-infected *Il1r1^−/−^* BMDCs (Fig. 5B), suggesting a minor involvement of IL-1RI signaling in BMDC apoptosis induction.

### Caspase-1/11-independent inflammasome activation and apoptosis induction in highly purified BMDCs

**Figure 6:**
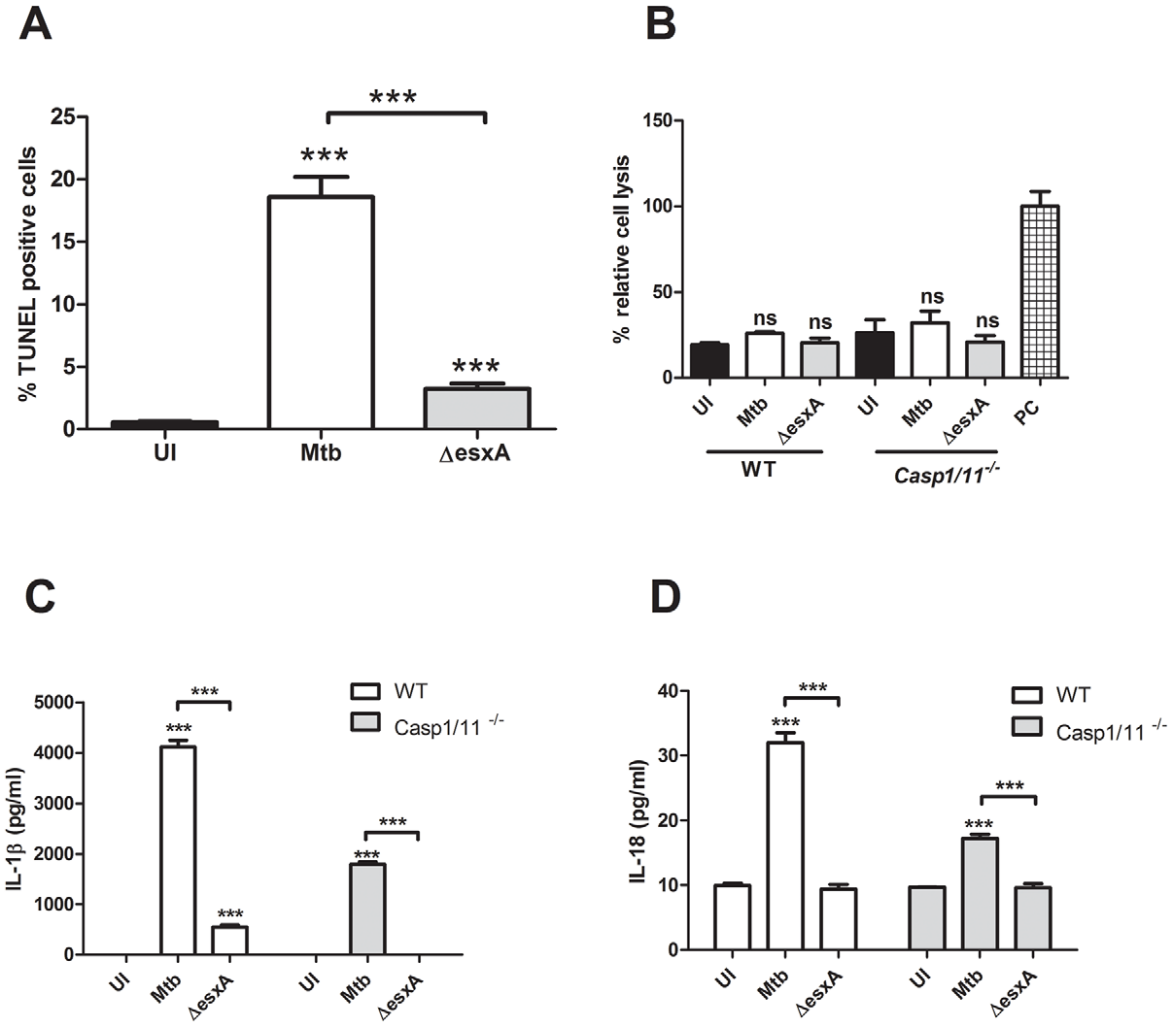
Highly purified CD11c^+^ dendritic cells confirmed the partially caspase-1/11 independent inflammasome activation upon infection with Mtb. CD11c^+^ sorted BMDCs from wild-type WT and *Casp1/11^−/−^* were left uninfected (UI, black bars), infected with Mtb (white bars) or the ΔesxA mutant (gray bars). After 24h the percentage of TUNEL positive cells was determined in (A). In (B) the amount of necrosis was determined by quantification of the release of adenylate kinase and normalized to the values obtained in detergent lysed cells (PC, striped bar). In (C) and (D) the same supernatants were analyzed for IL-1β and IL-18 secretion, respectively.

In our hands the unsorted population of cytokine derived BMDCs is on average >75% positive for DC cell surface markers (data not shown). In order to confirm that the ESX-1-dependent apoptosis induction and the caspase-1/11-independent inflammasome activation are not caused by the ∼25% of undefined cells we repeated key experiments using highly purified (>99% CD11c^+^) BMDCs (Fig. 6). First, we confirmed that the apoptosis induction in BMDCs is highly dependent upon the presence of functional ESX-1 secretion system, since the *esxA* mutant induced about 5fold less apoptosis compared to wild-type Mtb (Fig. 6A). Importantly, the observed caspase-1/11-independent induction of IL-1β secretion could also be confirmed in the sorted BMDCs and was also observed for IL-18 cytokine secretion (Fig. 6C+D). The differences in IL-1β and IL-18 secretion are not due to accidental release of immature cytokines since the amount of cell lysis was similar for all of the experimental conditions and cell types except for the positive control (Fig. 6B). We also could not detect any pro-IL-1β in the supernatants using pro-IL-1β specific ELISA (data not shown).

## Discussion

Dendritic cells (DCs) are antigen presenting cells that have a major role in initiating an adaptive immune response via priming of naïve T cells [40]. The role of DCs in host immunity to Mtb infections has become evident since their depletion in the mouse model of tuberculosis led to a delay in specific T cell activation and exacerbation of disease [41]. Furthermore, DCs serve as host cells in Mtb-infected lungs and are important for secretion of IL-12 and T cell priming [28], [29], [31], [42], [43]. The type VII secretion system of mycobacteria, ESX-1, secretes proteins that mediate a wide array of responses in infected macrophages [44], [45]. Here we determined that infected BMDCs induce strong IL-6 and TNF secretion independently of the presence of functional ESX-1 (Fig. 1A). This was consistent with the findings that the transcriptional response to induce IL-6 and TNF is not affected by the presence of ESX-1 in BMDCs [46]. Thioglycolate-induced peritoneal macrophages also induce strong TNF and IL-6 responses independent of the ESX-1 system [35]. In contrast, the inflammasome activation in BMDCs, as measured by IL-1β secretion and caspase-1 activity, is strongly dependent on the ESX-1 system (Fig. 1B-D) which is comparable to the reaction of BMDMs [22], [35]. Nevertheless, after 4 h and 8 h post infection the decrease in IL-1β secretion by *esxA* mutant infected cells is not only due to a lack in inflammasome activation but also to a decrease in pro-IL-1β production as determined by western blotting (Fig. 1E). Caspase-1 can augment TLR-mediated TNF and IL-6 production via cleavage of the TLR adapter protein TIRAP [47], [48]. The ESX-1 Mtb mutant does not activate caspase-1 efficiently in BMDCs as demonstrated by a lack of IL-1β secretion and FLICA substrate cleavage assay (Fig. 1C-D) but at the same time the mutant is not deficient in TNF and IL-6 and thus it seems that at least in murine BMDCs caspase-1/11 are not necessary for full induction of these proinflammatory cytokines.

Some pathogens induce pyronecrosis and/or pyroptosis upon infection of host cells [4], [49]. We investigated if either of these cell death pathways is induced in DCs after infection with Mtb. There was no reduction of apoptosis in ASC and Casp-1/11 deficient mice indicating that no pyroptosis was induced (Fig. 4B). There was no induction of necrosis either during the observed time period of 24 h (Fig. 4C) and consequently no pyronecrosis. Interestingly, it seems that Mtb induces a higher rate of apoptosis in DCs than what we have observed before when infecting BMDMs at identical MOI for 4 h [50]. Mtb has mechanisms to inhibit host cell apoptosis [12]. Nevertheless, it is an advantage for the host organisms if the host cell becomes apoptotic because macrophages from susceptible mouse strains undergo necrotic-type of cells death whereas macrophages from a resistant strain induce apoptosis [51]. It is not possible for Mtb to completely abolish the macrophage apoptosis response and hence one always observes an increase of apoptosis in Mtb infected cells when compared to uninfected cells. One could hypothesize that dendritic cells are more sensitive to undergoing apoptosis upon Mtb infection when compared to BMDMs. This could potentially be due to the higher rate of activity of their NOX2 system [52]. We previously demonstrated in murine macrophages that prolonged NOX2 activity and phagosomeal ROS accumulation caused apoptotic cell death [50].

In BMDMs and the human macrophage-like THP-1 cell line, it has recently been demonstrated that Mtb-induced IL-1β cytokine secretion is dependent upon the presence of NLRP3, ASC and Caspase-1 proteins [20], [22], [23], [24]. Here we show that the same inflammasome components are important for dendritic cell mediate IL-1β release upon Mtb infection (Fig. 3). Our results on the importance of NLRP3 are also consistent with the recent report that BMDCs of *Nlrp3^−/−^* mice do not induce IL-1β secretion after Mtb infection [32]. We furthermore excluded the contribution of NLRC4 and NOD2 in inflammasome activation (Fig. 3A and Fig. 3C). The latter result was of interest because NOD2 has been shown to be necessary for the induction of proinflammatory cytokines (TNF, IL-6 and IL-12) in BMDCs [53] and primary murine, alveolar macrophages [54]. Furthermore, NOD2 has been shown to associate with NLRP1 to induce caspase-1 activation [55], and NOD2 is also implicated in the muramyl dipeptide induced ASC/NLRP3 inflammasome activation [56]. Thus it was important to exclude the contribution of this NLR in Mtb-induced inflammasome activation in BMDCs in our study. In contrast, primary monocytes derived from human patients deficient in NOD2 activity, showed a decrease of IL-1β secretion after Mtb infection when compared to monocytes of healthy donors [39]. This difference could be accounted for by the fact that monocytes are quite distinct from macrophages and dendritic cells when it comes to IL-1β secretion since they have a constitutively active caspase-1 and thus do not require inflammasome activation for maturation of pro-IL-1β [26]. Another explanation could be the difference between human and murine cells used in these respective studies.

Interestingly, we demonstrated a small but significant level of IL-1β and IL-18 secretion that was independent of caspase-1/11 in BMDCs but still dependent upon ASC and NLRP3 (Fig. 3A+B,6C+D). This was unexpected since in all of the published results on macrophages the IL-1β maturation is completely dependent on the presence of caspase-1 [20], [23], [24]. One possibility is that other non-inflammatory caspases, such as caspase-8 or -9, are capable of cleaving pro-IL-1βand pro-IL-18 in the caspase-1/11 deficient murine BMDCs. It is not without precedence for caspases to have multiple functions [57]; for example, caspase-8 and 3/7 are important for activation of macrophages in the central nervous system without causing apoptosis [58]. Alternatively, mature IL-1β and IL-18 could be generated in a caspase-independent way as described to date mainly for neutrophils via cleavages by serine proteases such as proteinase-3, elastase or cathepsin-G [27]. This seems unlikely because these proteases are not dependent upon ASC for activation. A third possibility is that Mtb releases a protease capable of cleaving IL-1β similar to the fungal pathogen *Candida albicans* [59]. Nevertheless, one would then also expect to see a caspase-1/11-independent IL-1β secretion in Mtb-infected macrophages, unless the expression of this hypothetical protease is regulated differently in macrophages and dendritic cells.

The IL-1β response is of great importance for host defense against Mtb infections [17], [18], [19], [20], [21]. Here we provided the first detailed analysis of the inflammasome activation of dendritic cells and demonstrated that Mtb-induced IL-1β and IL-18 secretion is dependent upon ASC and NLRP3 but only partially on caspase-1/11.

## Materials and Methods

All animal studies were approved by the Institutional Care and Use Committee of The University of Maryland at College Park and were conducted in accordance with the IACUC guidelines and the National Institutes of Health Guide for the Care and Use of Laboratory Animals. Protocol# R-09-35.

### Mice

C57BL/6 wild-type, and *Nod2^−/−^,* mice were obtained from The Jackson Laboratories. *Casp1^−/−^*, *Nlrc4^−/−^, Nlrp3^−/−^*, *Asc^−/−^* and *Il1r1^−/−^* mice have been described elsewhere and are all in the C57BL/6 background [60], [61], [62]. Mice were maintained under pathogen-free conditions and used between 6 to 12 weeks of age.

### Cell culture

The generation of bone marrow-derived dendritic cells (BMDCs) method was performed as described [43] and consistently generated more than 75% of cells positive for CD11c and CD86 in our hands. Bone marrow cells were obtained from the femurs and tibia of mice. Cells were then cultured in DMEM supplemented with Penicillin (100 U/ml), Streptomycin (100 μg/ml), 2-mercaptoethanol (50 μM) (all from Invitrogen), 10% heat-inactivated FCS (Hyclone), and 200U/ml GM-CSF (Peprotech) on 100 mm dishes for 7–9 days. In order to confirm that BMDC and not minor contaminating cell populations are responsible for caspas-1-independent inflammasome activation FACS was used to purify 99% CD11c positive cells. Briefly, after GM-CSF differentiation on day 7, CD11c-positive BMDCs were purified by incubated with antibodies to block Fc-Receptors (clone 24G2) for 20 minutes at 4°C, then cells were stained with anti-mouse CD11c –PE clone N418 (cat# 12-0114-81) and anti-hamster IgG-PE. (cat# 12-4112-83) as isotype control for another 20 minutes at 4°C (both from eBioscience). Cells were washed twice in PBS and CD11c positive cells were isolated by cell sorting using the FACSAria (BD Bioscience) in the MPRI flow cytometry core facility. The resulting sorted cell population is 99% positive for CD11c.

### Bacteria

*M. tuberculosis* H37Rv (ATCC 25618) was obtained from Dr. W.R. Jacobs Jr. (AECOM), MtbΔesxA was kindly provided by Dr. Lian-Yong Gao (University of Maryland). All mycobacteria were grown in 7H9 media (Invitrogen) supplemented with 0.05%glycerol (Sigma), 0.05% TWEEN-80 (Sigma), and 10% ADC (Invitrogen). For selective media 50μg/ml hygromycin (Invitrogen) was added.

### Cell culture and infection

Cultured medium containing differentiated BMDCs in suspension was collected and centrifuged. Cell pellets were then re-suspended in infection media containing only GM-CSF. Bacteria were grown to an OD_600_ ranging from 0.5 to 0.8 and prepared as described. Infections were carried out at a multiplicity of infection (MOI) of 10∶1 for 4 hours in infection media containing 10% non-heat inactivated FCS. After 4 hours, extracellular bacteria were removed by 2 washes with PBS and the cells were incubated in chase media containing GM-CSF and 100 μg/ml of gentamicin (Invitrogen).

### Cell death and caspase activation assays

The TUNEL assay was performed using the “In Situ Cell Death Detection Kit-Fluorescein or –TMR Red” (Roche Applied Sciences). The assay was carried out as described by the manufacturer and the percentage of stained cells was analyzed using flow cytometry.

Caspase 3/7 or caspase-1 activation was detected using the FLICA-caspases-3-7- or FLICA-caspase-1 assays (ImmunoChemistry Technologies, Cat # 94 or Cat#97, respectively). Cells were infected and harvested at indicated timepointspost infection. Assay was performed according to manufacture protocol by staining the cells with FLICA reagent for 1h at 37°C at under 5% CO_2_ then washed before analysis by flow cytometry.

The adenylate kinase (AK) release assay, Toxilight®BioAssay (Lonza, Cat # LT07-217) was used to quantify necrotic cell death. The assay was performed according to the manufacturer's instructions using cell free supernatants harvested 24 hours post infection.

### Cytokine Assays

The BD™ CBA Kit (BD Biosciences, Cat # 552364) was used to measure Interleukin-6 (IL-6), Interleukin-10 (IL-10), MCP-1, Interferon-γ (IFN-γ) and TNF protein levels in a single sample. The assay was performed according to manufacturer's instruction. Data was collected using a flow cytometer and analyzed with the FCAP Array™ Software.

ELISA was used to measure secreted IL-1β, pro-IL1β and IL-18 in cell supernatants collected 24 h post infection using BD OptEIA^TM^ Set Mouse IL-1β kit (BD Biosciences cat# 559603), mouse IL-1 beta Pro-form Ready-SET-Go! ELISA Set (eBioscience #cat. 88-8014-88 ) and Platinum Mouse IL-18 kit (eBioscience #BMS618/2), respectively.

### Western Blotting

BMDCs were seeded at a density of 0.75×10̂6 cells per well in 24 well plates and infected at an MOI of 10 for four hours. After which the extracellular bacteria were washed away and the cells were either harvested or incubated with fresh media for the indicated time points. For cell lysis, washed cells were resuspended in 300µl of 1x lysis buffer (50mM Tri-HCl pH 8.0, 5mM EDTA, 150mM NaCl, 1% Triton X-100) suplemented with EDTA-free protease inhibitor cocktail ( Roche #11836170001), vortexed and placed on iced for 5 mins. The lysed cells were centrifuged at 16,000g for 30mins at 4°C to obtain the post-nuclear supernatant (cell lysate). 10ug of cell lysates mixed with 4x sample loading buffer were separated on 4–20% SDS-PAGE gels and transferred to PVDF membranes. The membrane was blocked with PBS/ 5% drymilk for 1 hour and then incubated overnight with the primary antibody. This was followed by incubation with HRP-conjugated secondary antibody for 1 hour. The membrane was then developed using the chemiluminescent pico substrate (Pierce #34078) and exposed on films (GeneMate #F-9023). The primary antibodies used were: Anti- Il-1β (R&D systems #AF401NA) at a concentration of 0.15µg/ml in 0.3% BSA and anti- GAPDH antibody (Cell Signaling #2118) at 1∶1000 dilution in PBS/5% drymilk. The secondary antibodies used were: Donkey anti-goat (Jackson #705-035-003) at 1∶25,000 and goat anti-rabbit (Jackson #111-035-003) at 1∶50,000 dilutions respectively.

### Statistical analysis

Statistical analysis was performed on at least three independent experiments using GraphPad Prism 5.0 software and One-way ANOVA with Tukey's post-test unless otherwise noted in the figure legends. Shown are representative results of triplicate values with standard deviation. The range of p-values is indicated as follows: * p = 0.01–0.5; ** p = 0.001–0.01 and *** p = 0.0001–0.001.

## References

[1] Pedra JH, Cassel SL, Sutterwala FS (2009). Sensing pathogens and danger signals by the inflammasome.. Curr Opin Immunol.

[2] Schroder K, Tschopp J (2010). The inflammasomes.. Cell.

[3] Ting JP, Lovering RC, Alnemri ES, Bertin J, Boss JM (2008). The NLR gene family: a standard nomenclature.. Immunity.

[4] Ting JP, Willingham SB, Bergstralh DT (2008). NLRs at the intersection of cell death and immunity.. Nat Rev Immunol.

[5] Bergsbaken T, Fink SL, Cookson BT (2009). Pyroptosis: host cell death and inflammation.. Nat Rev Microbiol.

[6] Fink SL, Cookson BT (2006). Caspase-1-dependent pore formation during pyroptosis leads to osmotic lysis of infected host macrophages.. Cell Microbiol.

[7] Chen Y, Smith MR, Thirumalai K, Zychlinsky A (1996). A bacterial invasin induces macrophage apoptosis by binding directly to ICE.. EMBO J.

[8] Hersh D, Monack DM, Smith MR, Ghori N, Falkow S (1999). The Salmonella invasin SipB induces macrophage apoptosis by binding to caspase-1.. Proc Natl Acad Sci U S A.

[9] Fink SL, Bergsbaken T, Cookson BT (2008). Anthrax lethal toxin and Salmonella elicit the common cell death pathway of caspase-1-dependent pyroptosis via distinct mechanisms.. Proc Natl Acad Sci U S A.

[10] Bergsbaken T, Cookson BT (2007). Macrophage activation redirects yersinia-infected host cell death from apoptosis to caspase-1-dependent pyroptosis.. PLoS Pathog.

[11] Dye C, Williams BG (2010). The population dynamics and control of tuberculosis.. Science.

[12] Briken V, Miller JL (2008). Living on the edge: inhibition of host cell apoptosis by Mycobacterium tuberculosis.. Future Microbiol.

[13] Pieters J (2008). Mycobacterium tuberculosis and the macrophage: maintaining a balance.. Cell Host Microbe.

[14] Russell DG (2007). Who puts the tubercle in tuberculosis?. Nat Rev Microbiol.

[15] Behar SM, Divangahi M, Remold HG (2010). Evasion of innate immunity by Mycobacterium tuberculosis: is death an exit strategy?. Nat Rev Microbiol.

[16] Philips JA, Ernst JD (2012). Tuberculosis pathogenesis and immunity.. Annu Rev Pathol.

[17] Sugawara I, Yamada H, Kaneko H, Mizuno S, Takeda K (1999). Role of interleukin-18 (IL-18) in mycobacterial infection in IL-18-gene-disrupted mice.. Infect Immun.

[18] Sugawara I, Yamada H, Hua S, Mizuno S (2001). Role of interleukin (IL)-1 type 1 receptor in mycobacterial infection.. Microbiol Immunol.

[19] Fremond CM, Togbe D, Doz E, Rose S, Vasseur V (2007). IL-1 receptor-mediated signal is an essential component of MyD88-dependent innate response to Mycobacterium tuberculosis infection.. J Immunol.

[20] Mayer-Barber KD, Barber DL, Shenderov K, White SD, Wilson MS (2010). Caspase-1 independent IL-1beta production is critical for host resistance to mycobacterium tuberculosis and does not require TLR signaling in vivo.. J Immunol.

[21] Schneider BE, Korbel D, Hagens K, Koch M, Raupach B (2010). A role for IL-18 in protective immunity against Mycobacterium tuberculosis.. Eur J Immunol.

[22] Koo IC, Wang C, Raghavan S, Morisaki JH, Cox JS (2008). ESX-1-dependent cytolysis in lysosome secretion and inflammasome activation during mycobacterial infection.. Cell Microbiol.

[23] McElvania Tekippe E, Allen IC, Hulseberg PD, Sullivan JT, McCann JR (2010). Granuloma formation and host defense in chronic Mycobacterium tuberculosis infection requires PYCARD/ASC but not NLRP3 or caspase-1.. PLoS One.

[24] Mishra BB, Moura-Alves P, Sonawane A, Hacohen N, Griffiths G (2010). Mycobacterium tuberculosis protein ESAT-6 is a potent activator of the NLRP3/ASC inflammasome.. Cell Microbiol.

[25] Dorhoi A, Nouailles G, Jorg S, Hagens K, Heinemann E (2011). Activation of the NLRP3 inflammasome by Mycobacterium tuberculosis is uncoupled from susceptibility to active tuberculosis.. Eur J Immunol.

[26] Netea MG, Nold-Petry CA, Nold MF, Joosten LA, Opitz B (2009). Differential requirement for the activation of the inflammasome for processing and release of IL-1beta in monocytes and macrophages.. Blood.

[27] Netea MG, Simon A, van de Veerdonk F, Kullberg BJ, Van der Meer JW (2010). IL-1beta processing in host defense: beyond the inflammasomes.. PLoS Pathog.

[28] Jiao X, Lo-Man R, Guermonprez P, Fiette L, Deriaud E (2002). Dendritic cells are host cells for mycobacteria in vivo that trigger innate and acquired immunity.. J Immunol.

[29] Wolf AJ, Linas B, Trevejo-Nunez GJ, Kincaid E, Tamura T (2007). Mycobacterium tuberculosis infects dendritic cells with high frequency and impairs their function in vivo.. J Immunol.

[30] Urdahl KB, Shafiani S, Ernst JD (2011). Initiation and regulation of T-cell responses in tuberculosis.. Mucosal Immunol.

[31] Blomgran R, Desvignes L, Briken V, Ernst JD (2012). Mycobacterium tuberculosis inhibits neutrophil apoptosis, leading to delayed activation of naive CD4 T cells.. Cell Host Microbe.

[32] Walter K, Holscher C, Tschopp J, Ehlers S (2010). NALP3 is not necessary for early protection against experimental tuberculosis.. Immunobiology.

[33] Kayagaki N, Warming S, Lamkanfi M, Vande Walle L, Louie S (2011). Non-canonical inflammasome activation targets caspase-11.. Nature.

[34] Master SS, Rampini SK, Davis AS, Keller C, Ehlers S (2008). Mycobacterium tuberculosis prevents inflammasome activation.. Cell Host Microbe.

[35] Kurenuma T, Kawamura I, Hara H, Uchiyama R, Daim S (2009). The RD1 locus in the Mycobacterium tuberculosis genome contributes to activation of caspase-1 via induction of potassium ion efflux in infected macrophages.. Infect Immun.

[36] Carlsson F, Kim J, Dumitru C, Barck KH, Carano RA (2010). Host-detrimental role of Esx-1-mediated inflammasome activation in mycobacterial infection.. PLoS Pathog.

[37] Stanley SA, Raghavan S, Hwang WW, Cox JS (2003). Acute infection and macrophage subversion by Mycobacterium tuberculosis require a specialized secretion system.. Proc Natl Acad Sci U S A.

[38] Hsu T, Hingley-Wilson SM, Chen B, Chen M, Dai AZ (2003). The primary mechanism of attenuation of bacillus Calmette-Guerin is a loss of secreted lytic function required for invasion of lung interstitial tissue.. Proc Natl Acad Sci U S A.

[39] Kleinnijenhuis J, Joosten LA, van de Veerdonk FL, Savage N, van Crevel R (2009). Transcriptional and inflammasome-mediated pathways for the induction of IL-1beta production by Mycobacterium tuberculosis.. Eur J Immunol.

[40] Steinman RM, Idoyaga J (2010). Features of the dendritic cell lineage.. Immunol Rev.

[41] Tian T, Woodworth J, Skold M, Behar SM (2005). In vivo depletion of CD11c+ cells delays the CD4+ T cell response to Mycobacterium tuberculosis and exacerbates the outcome of infection.. J Immunol.

[42] Bodnar KA, Serbina NV, Flynn JL (2001). Fate of Mycobacterium tuberculosis within murine dendritic cells.. Infect Immun.

[43] Hickman SP, Chan J, Salgame P (2002). Mycobacterium tuberculosis induces differential cytokine production from dendritic cells and macrophages with divergent effects on naive T cell polarization.. J Immunol.

[44] Abdallah AM, Gey van Pittius NC, Champion PA, Cox J, Luirink J (2007). Type VII secretion – mycobacteria show the way.. Nat Rev Microbiol.

[45] Simeone R, Bottai D, Brosch R (2009). ESX/type VII secretion systems and their role in host-pathogen interaction.. Curr Opin Microbiol.

[46] Majlessi L, Brodin P, Brosch R, Rojas MJ, Khun H (2005). Influence of ESAT-6 secretion system 1 (RD1) of Mycobacterium tuberculosis on the interaction between mycobacteria and the host immune system.. J Immunol.

[47] Li P, Allen H, Banerjee S, Franklin S, Herzog L (1995). Mice deficient in IL-1 beta-converting enzyme are defective in production of mature IL-1 beta and resistant to endotoxic shock.. Cell.

[48] Miggin SM, Palsson-McDermott E, Dunne A, Jefferies C, Pinteaux E (2007). NF-kappaB activation by the Toll-IL-1 receptor domain protein MyD88 adapter-like is regulated by caspase-1.. Proc Natl Acad Sci U S A.

[49] Mariathasan S, Monack DM (2007). Inflammasome adaptors and sensors: intracellular regulators of infection and inflammation.. Nat Rev Immunol.

[50] Miller JL, Velmurugan K, Cowan MJ, Briken V (2010). The Type I NADH Dehydrogenase of Mycobacterium tuberculosis Counters Phagosomal NOX2 Activity to Inhibit TNF-α-Mediated Host Cell Apoptosis.. PLoS Pathog 6.

[51] Pan H, Yan BS, Rojas M, Shebzukhov YV, Zhou H (2005). Ipr1 gene mediates innate immunity to tuberculosis.. Nature.

[52] Mantegazza AR, Savina A, Vermeulen M, Pérez L, Geffner J (2008). NADPH oxidase controls phagosomal pH and antigen cross-presentation in human dendritic cells.. Blood.

[53] Gandotra S, Jang S, Murray PJ, Salgame P, Ehrt S (2007). Nucleotide-binding oligomerization domain protein 2-deficient mice control infection with Mycobacterium tuberculosis.. Infect Immun.

[54] Divangahi M, Mostowy S, Coulombe F, Kozak R, Guillot L (2008). NOD2-deficient mice have impaired resistance to Mycobacterium tuberculosis infection through defective innate and adaptive immunity.. J Immunol.

[55] Hsu LC, Ali SR, McGillivray S, Tseng PH, Mariathasan S (2008). A NOD2-NALP1 complex mediates caspase-1-dependent IL-1beta secretion in response to Bacillus anthracis infection and muramyl dipeptide.. Proc Natl Acad Sci U S A.

[56] Pan Q, Mathison J, Fearns C, Kravchenko VV, Da Silva Correia J (2007). MDP-induced interleukin-1beta processing requires Nod2 and CIAS1/NALP3.. J Leukoc Biol.

[57] Kuranaga E (2012). Beyond apoptosis: caspase regulatory mechanisms and functions in vivo.. Genes Cells.

[58] Burguillos MA, Deierborg T, Kavanagh E, Persson A, Hajji N (2011). Caspase signalling controls microglia activation and neurotoxicity.. Nature.

[59] Beausejour A, Grenier D, Goulet JP, Deslauriers N (1998). Proteolytic activation of the interleukin-1beta precursor by Candida albicans.. Infect Immun.

[60] Adachi O, Kawai T, Takeda K, Matsumoto M, Tsutsui H (1998). Targeted disruption of the MyD88 gene results in loss of IL-1- and IL-18-mediated function.. Immunity.

[61] Mariathasan S, Newton K, Monack DM, Vucic D, French DM (2004). Differential activation of the inflammasome by caspase-1 adaptors ASC and Ipaf.. Nature.

[62] Sutterwala FS, Ogura Y, Szczepanik M, Lara-Tejero M, Lichtenberger GS (2006). Critical role for NALP3/CIAS1/Cryopyrin in innate and adaptive immunity through its regulation of caspase-1.. Immunity.

